# Nonlinear Analysis of Rotational Springs to Model Semi-Rigid Frames

**DOI:** 10.3390/e24070953

**Published:** 2022-07-09

**Authors:** César Antonio Rodríguez González, Julio José Caparrós-Mancera, José Antonio Hernández-Torres, Ángel Mariano Rodríguez-Pérez

**Affiliations:** Department of Mining, Mechanical, Energy and Construction Engineering, University of Huelva, 21004 Huelva, Spain; julio.caparros@diesia.uhu.es (J.J.C.-M.); joseantonio.hernandez@dimme.uhu.es (J.A.H.-T.); angel.rodriguez@dci.uhu.es (Á.M.R.-P.)

**Keywords:** stiffness matrix, P-delta effect, handle function, Newton–Raphson

## Abstract

This paper explains the mathematical foundations of a method for modelling semi-rigid unions. The unions are modelled using rotational rather than linear springs. A nonlinear second-order analysis is required, which includes both the effects of the flexibility of the connections as well as the geometrical nonlinearity of the elements. The first task in the implementation of a 2D Beam element with semi-rigid unions in a nonlinear finite element method (FEM) is to define the vector of internal forces and the tangent stiffness matrix. After defining the formula for this vector and matrix in the context of a semi-rigid steel frame, an iterative adjustment of the springs is proposed. This setting allows a moment–rotation relationship for some given load parameters, dimensions, and unions. Modelling semi-rigid connections is performed using Frye and Morris’ polynomial model. The polynomial model has been used for type-4 semi-rigid joints (end plates without column stiffeners), which are typically semi-rigid with moderate structural complexity and intermediate stiffness characteristics. For each step in a non-linear analysis required to adjust the matrix of tangent stiffness, an additional adjustment of the springs with their own iterative process subsumed in the overall process is required. Loops are used in the proposed computational technique. Other types of connections, dimensions, and other parameters can be used with this method. Several examples are shown in a correlated analysis to demonstrate the efficacy of the design process for semi-rigid joints, and this is the work’s application content. It is demonstrated that using the mathematical method presented in this paper, semi-rigid connections may be implemented in the designs while the stiffness of the connection is verified.

## 1. Introduction

The use of semi-rigid joints in structural systems has a variety of engineering applications. Given its speed of assembly, one of its applications is for circumstances when it is interesting for constructive reasons as seismic studies and protection [1,2]. When the connections are properly built, experimental tests have shown that semi-rigid steel frames exhibit ductile and stable hysteretic behavior [3,4]. Hysteretic loops with semi-rigid connections, one of the key damping sources of structures, disperse the energy [5]. The issue is figuring out how to simulate these semi-rigid joints, which behave differently from recessed joints. Its behaviour, as evidenced by experimentally validated models, already reveals a lack of linearity in the behaviour of these joints. The resolution of the case using an incremental method is only one element of the heuristic: the fundamental difficulty is in modelling the union when the stiffness matrix is not constant throughout the process. In the context of the idea of a tangent stiffness matrix, or simply a tangent matrix, in a non-linear finite element method (FEM), some previous works are still relevant [6,7,8].

It is essential to know the work of Dhillon, B.S. and O’Malley, J.W., III in terms of their application to beams with semi-rigid connections at the ends and a mathematical formulation for point-to-point load [9]. This includes the tangent matrix expression while taking the P-delta effect into account. [10]

This work contains a model of semi-rigid joints created using non-linear rotational springs. Other authors have already addressed this topic, with a focus on mathematical aspects in some cases [11,12,13,14,15].

The novelty of this work is the mathematical formulation for spring adjustment using an incremental control method in weight with two Newton–Raphson in series, each anointed with a control or governance method for the structure. Its application in the field of engineering appears when it is necessary to calculate frames built with steel sections. Although the case is specific to a certain type of semi-rigid union (type-4 end plate without column stiffeners, from the model of Fryes and Morris [16], implemented, among others, by Degertekin, S. and Hayalioglu, M. [10]), it is completely extrapolated to other structural systems [17].

The model’s employment necessitates the resolution of two fundamental questions. To begin, the internal vector of forces and the elemental tangent matrix are defined. In this case, the non-linearity of the springs is taken into account, as they have non-constant stiffnesses and a non-linear relationship between the giro and moment. Second, the P-delta effect is considered, which has already been studied by other authors and is used often in the structural and mechanical engineering fields [18]. This effect was taken into account when determining the tangent stiffness matrix. The cases studied in this work concern the use of type-4 semi-rigid connections (end plate without column stiffeners) in various boundary conditions. In terms of the use of an FEM implemented in the appropriate code, it is not linear to be able to address nonlinear incremental method resolution. Details about the FEM’s non-linear formulation can be found in various works [19,20,21]. In this case, the beam element is a non-linear Beam2D consisting of a single element. Its assembly into structures with more finite elements can be approached from the fundamental element. Although this base is required, it is not the focus of this work, which focuses on the modelling of a Beam2D element with semi-rigid joints at its extremities. On the other hand, due to their importance, a review of the evolution of this type of connection’s model is conducted [22,23,24].

## 2. Materials and Methods

### 2.1. Problem-Solving Strategy

The topic is the adjustment of rotational springs to model a Beam2D element with semi-rigid connections at its extremities. Adjusting the springs of a Beam2D element requires defining a non-linear relationship between rotation and time. Although this topic has been addressed in a broad sense, its resolution using an incremental method, which is useful for analysis in a non-linear FEM environment, is a case of interest [25,26].

To solve this problem, a Newton–Raphson analogue method has been used in conjunction with a general control method because each step of loading (or loading and displacement, depending on the general control method used) causes a rotation in the spring, which causes an update of the bending moments at the extremities. An expression will be obtained in which time and motion are linked. The springs as well as the stiffness of the section influence this expression. Nested loops are used to solve the problem and codify it. To model the behaviour of springs, it is essential to understand the variables that define their behaviour. In reality, they correspond to the type of semi-rigid union that should be used. The polynomial model of Fryes and Morris for type-4 unions defines the behaviour of these springs. Other polynomial models, or even non-polynomial models, can be used for other types of connections but only with the caution of performing the appropriate derivation. 

On the other hand, in order to implement it with an informatic tool and the corresponding code in the context of a nonlinear FEM, some works have been revised [19,20,21], as well as others in which nonlinear problems are addressed in various contexts [13,27]. For a stability failure or post-peak behaviour, the arc-length method with load and displacement control is necessary. However, for a nonlinear analysis between moment and rotation, before the formation of point plastic hinges, the Newton–Raphson method is sufficient [10].

### 2.2. Obtaintion of the Vector of Internal Forces from Its Analytic Expression

In the context of a nonlinear FEM, which is appropriate for modelling semi-rigid joints in Beam2D elements, the expression that defines the elemental vector of forces must be resolved first. This has been carried out taking into account the second-order effect P, which refers to the transverse deformations caused by the axils. In terms of loads, it will be limited to continuous load, yet same analysis can also be used to punctual load [9]. In any case, in an FEM formulation, the loads, whether punctual, continuous, or a combination of both, will be transferred to the nodes of the beam’s extremes using the tools from vectorial mechanics, so that all effects and the nodal loads are equivalent to the first. The steps for the resolution are:

(1) Composition and resolution of the free-body diagram of a semi-rigid beam without the P effect, as well as the determination of bending moments and strains;
(2) Composition and resolution of the free-body diagram of a semi-rigid beam including the P effect, and the indirect methodology to include the strains in the forces vector from the stiffness matrix;
(3) Define the vector of internal forces according to one or more element displacements and a recalculation of the vector of forces based on the results of point (2) using the elemental stiffness matrix.

**Figure 1:**
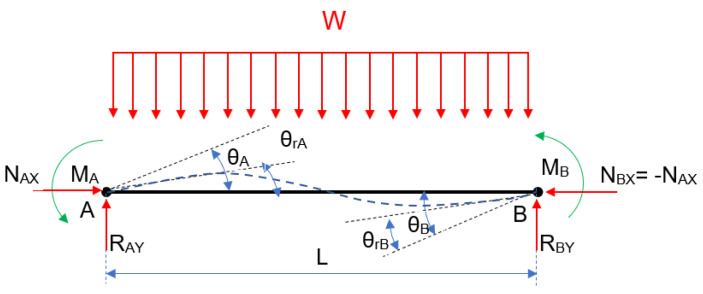
Free−body diagram with a constant load W.

**Figure 2:**
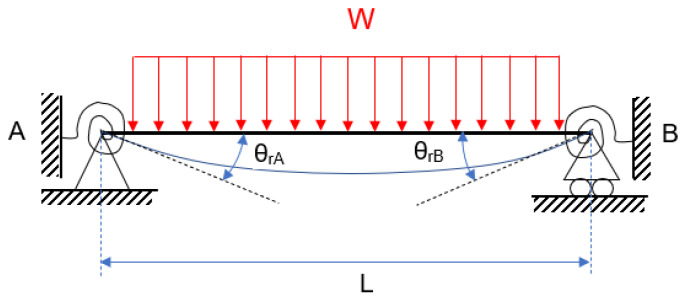
Semi-rigid beam with a continuous load.

Without the P effect, Figure 1 is obtained:

The bending moments $M_{A}$ and $M_{B}$ are in charge of recording both the continuous load effect and the opposite rotation effect provided by the springs. Following that, Figure 2 shows a semi-rigid beam with a continuous load.

Here, L is the beam’s length, and $\theta_{rA}$ and $\theta_{rB}$ are the semi-rigid beam’s rotation at their respective ends, A and B. These rotations are due to the deflexion caused by the corresponding load, which is amplified by the effect of both springs at their ends. The semi-rigid nature of the beam necessitates a flawless fit at its ends. The rotational springs, not the lineal ones, will modulate the beam’s behaviour between semi-rigid and embedded, depending on the used type of semi-rigid union.

**Figure 3:**
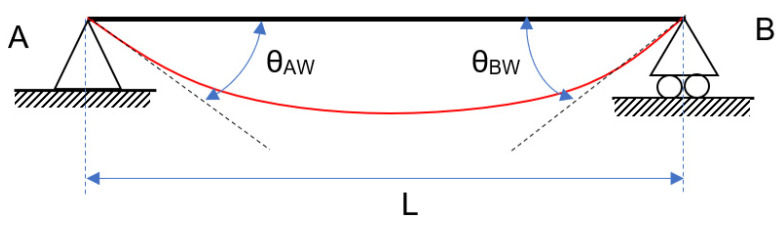
Semi-rigid beam: generated by a continuous load.

**Figure 4:**
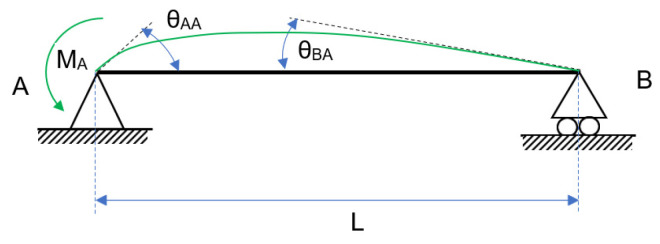
Semi-rigid beam: generated by the spring in A and M_A_.

**Figure 5:**
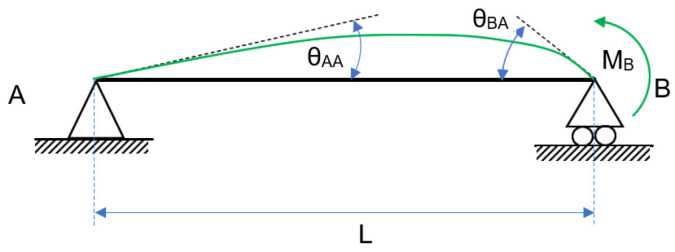
Semi-rigid beam: generated by the spring in B and M_B_.

The effect of the springs opposes the effect of the external loads, causing a rotation in the opposite direction. In Figure 3, Figure 4 and Figure 5, the figure for continuous loading becomes smudged. The diagram’s springs are removed and replaced by their effects at the appropriate moment and rotation. Each diagram represents an independent phenomenon. Figure 3 depicts the effect of the loads on the rotation, whereas Figure 4 and Figure 5 illustrate the moment and rotation caused by non-linear springs. Each spring will have an impact on the entire beam, regardless of which one is used. When the principle of superposition is applied, an equivalence is obtained that allows the two effects to be compared.

The final step entails replacing moment and rotation in each of the extremes to obtain the equations with the moments that will be used in subsequent reasoning. It is important to note that the loads will not generate in any case a moment of underrun; however, they will generate a rotation, and it is necessary to calculate it based on these loads. This rotation causes a deflection, which is partially counteracted by the springs. Extending Euler–Bernoulli’s beam theory to the prismatic and constant curvature-curved beam, the moment–curvature relation can be stated as in Equation (1):

(1) $$\theta_{rA}=\frac{M_{A}}{k_{A}}\;\;\;\;\;\;\;\;\;\;\;\;\;\;\;\;\;\;\;\;\;\;\theta_{rB}=\frac{M_{B}}{k_{B}}$$

where:

$\theta_{ri}$: The effect of the springs on the relative rotation of the beam in *i* depend, of the Frye and Morris’ polynomial model for type-4 unions;

$k_{i}:$ Rotational stiffness of the spring in the end *i*;

$M_{i}:$ Rotation moment in the extreme *i*.

The following rotations are obtained by removing the springs at the extremes and considering the continuous load, W, the $M_{A}$ and $M_{B}$ moments, and establishing the rotation in an elastic plan. Equation (2) is obtained:

(2) $$\theta_{AW}=\frac{W\cdot L^{3}}{6\cdot E\cdot I}\;\;\;\;\;\;\;\;\;\;\;\;\;\;\;\;\;\;\;\;\;\;\theta_{BW}=-\frac{W\cdot L^{3}}{6\cdot E\cdot I}$$

It is worth noting that the bending caused by a non-linear spring in a semi-rigid beam has been replaced by an equivalent bending moment applied at the extreme. This moment generates two rotations: one larger at the extreme where the moment is applied and one smaller at the opposite extreme. Equations (3) and (4) are obtained by using the beam symmetry:

(3) $$\theta_{AA\left(MA\right)}=-\frac{M_{A}\cdot L}{3\cdot E\cdot I}\;\;\;\;\;\;\;\;\;\;\;\;\;\;\;\;\;\;\;\;\;\;\theta_{BA\left(MA\right)}=\frac{M_{A}\cdot L}{6\cdot E\cdot I}$$

(4) $$\theta_{AB\left(MB\right)}=\frac{M_{B}\cdot L}{3\cdot E\cdot I}\;\;\;\;\;\;\;\;\;\;\;\;\;\;\;\;\;\;\;\;\;\;\theta_{B\left(MB\right)}=-\frac{M_{B}\cdot L}{6\cdot E\cdot I}$$

Adding the three rotations at either end, Equation (5) is obtained:

(5) $$\theta_{rA}=\theta_{AW}+\theta_{AA}+\theta_{AB}\;\;\;\;\;\;\;\;\;\;\;\;\;\;\;\theta_{rB}=\theta_{BW}+\theta_{BB}+\theta_{BA}$$

Knowing the rotations in a semi-rigid beam due to a constant load and two moments at either end that will replace the springs, it is possible to establish equations that relate moment and global rotation. Equations (6) and (7) are obtained by combining Equations (3)–(5) into one and rearranging the terms:

(6) $$\frac{M_{A}}{k_{A}}=\frac{W\cdot L^{3}}{6\cdot E\cdot I}--\frac{M_{A}\cdot L}{3\cdot E\cdot I}-\frac{M_{B}\cdot L}{6\cdot E\cdot I}$$

(7) $$\frac{M_{B}}{k_{B}}=\frac{W\cdot L^{3}}{6\cdot E\cdot I}--\frac{M_{B}\cdot L}{3\cdot E\cdot I}-\frac{M_{A}\cdot L}{6\cdot E\cdot I}$$

Equations (8) and (9) are generated by rearranging these equations and defining variables that depend on the spring stiffness and material constants:

(8) $$2\cdot M_{A}\cdot \left(1+3\cdot actA\right)+M_{B}=2\cdot M_{A\left(W\right)}+M_{B\left(W\right)}$$

(9) $$2\cdot M_{B}\cdot \left(1+3\cdot actB\right)+M_{A}=2\cdot M_{B\left(W\right)}+M_{A\left(W\right)}$$

where:

$M_{A}:$ Value of the moment in *A*, updated because of the springs effect;

$M_{B}:$ Value of the moment in *B*, updated because of the springs effect;

$M_{A\left(W\right)}:\;$ Value of the moment in *A* generated because of a continuous load;

$M_{B\left(W\right)}:$ Value of the moment in *B* generated because of a continuous load;

actA: Updated variable in the *A* moment due to the effect of spring *A*. Its value is *E·I*/(*L·kA*);

actB: Updated variable in the *B* moment due to the effect of spring *A*. Its value is *E·I*/(*L·kB*);

Coupling the moments in *A* and *B* according to Equations (1) and (5) and Equations (6) and (7), Equations (10) and (11) are obtained:

(10) $$M_{A}=\frac{M_{A\left(W\right)}+6\cdot actB\cdot M_{B\left(W\right)}}{\left(1+4\cdot actA+4\cdot actB+12\cdot actA\cdot actB\right)}$$

(11) $$M_{B}=\frac{M_{B\left(W\right)}+6\cdot actA\cdot M_{A\left(W\right)}}{\left(1+4\cdot actB+4\cdot actA+12\cdot actA\cdot actB\right)}$$

Finally, a relationship between the moment and rotation is found, which is reliant not only on the stiffness of the section but also on the stiffness of the springs. The values at the ends due to the load are those referred to in Equation (12) in terms of shear forces, taking into account equilibrium conditions and the values at the ends due to the load:

(12) $$V_{A\left(W\right)}=\frac{W\cdot L}{2}\;\;\;\;\;\;\;\;\;\;\;\;\;\;\;\;\;\;\;\;\;\;V_{B\left(W\right)}=\frac{W\cdot L}{2}$$

Following the same reasoning and procedure, we arrive at Equations (13) and (14):

(13) $$V_{A}=\frac{V_{A\left(W\right)}+6\cdot actB\cdot V_{B\left(W\right)}}{\left(1+4\cdot actA+4\cdot actB+12\cdot actA\cdot actB\right)}$$

(14) $$V_{B}=\frac{V_{B\left(W\right)}+6\cdot actA\cdot V_{A\left(W\right)}}{\left(1+4\cdot actB+4\cdot actA+12\cdot actA\cdot actB\right)}$$

where:

$V_{A}$: Value of the shear forces in *A*, updated because of the effect of the springs;

$V_{B}$: Value of the shear forces in *B*, updated because of the effect of the springs.

The *actA* and *actB* variables update the springs’ effect and are only dependent on spring-specific variables (stiffness) and material constants (*E*, *I*). According to Frye and Morris’ polynomial model, the stiffness value is dependent on associated parameters. Convergence issues can arise if you make the wrong decision. Studies on this topic may be found in Benterkia’s thesis [28] and Diaz et al.’s review of many models [29].

**Figure 6:**
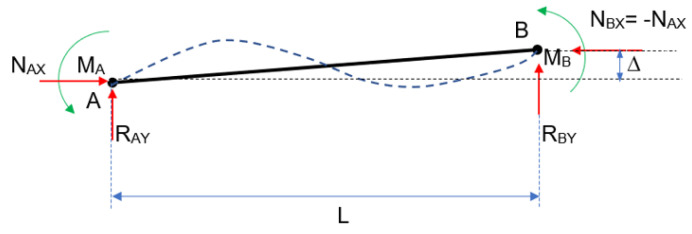
Semi-rigid beam: P-delta effect (I).

**Figure 7:**
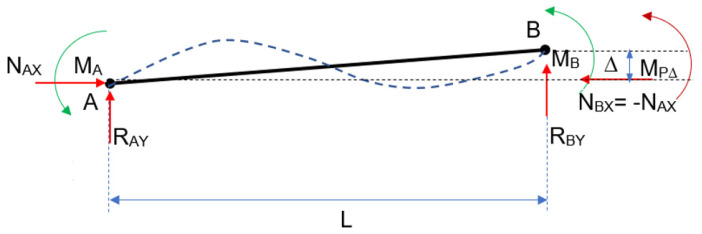
Semi-rigid beam: P-delta effect (II).

Analysing the effect transversal displacement in the beam as shown in Figure 6: 

In this scenario, Figure 7, the axial forces are no longer perfectly aligned, and a second order moment appears:

Whether there are axial forces of traction or compression impacting the transverse deformation, the moment mentioned appears. The P effect is actually the combined influence of the axial and the moment on the transverse deformation caused by the first. As a result, although in a nonlinear manner, this axial force indirectly affects the tangent stiffness matrix. Its impact can be felt in this stiffness test, as we will see below. The action of loads and springs is reinforced in this situation by the transverse displacement, caused by the axials’ influence. There are a number of arguments that can be used to bring the solution closer together. 

Returning to the case of a simple-supported beam with two rotational springs at its ends, if the springs and their respective rigidities are included (Equation (1)), it can be demonstrated how this affects the transverse deformation quantitatively, allowing modified slope deflection equations to be used. These equations are derived from the equations already inferred above, while also accounting for the fact that the springs change the rotation (Equations (4) and (5)) from $\theta_{A}$ and $\theta_{B}$ to $\theta_{A}$ − $\theta_{rA}$ and $\theta_{B}$ − $\theta_{rB}$, respectively. Equations (15) and (16) are the outcome of this:

(15) $$M_{A}=\frac{E\cdot I}{L}\left[4\cdot \left(\theta_{A}-\frac{M_{A}}{k_{A}}\right)+2\cdot 4\cdot \left(\theta_{B}-\frac{M_{B}}{k_{B}}\right)\right]$$

(16) $$M_{B}=\frac{E\cdot I}{L}\left[4\cdot \left(\theta_{B}-\frac{M_{B}}{k_{B}}\right)+2\cdot 4\cdot \left(\theta_{A}-\frac{M_{A}}{k_{A}}\right)\right]$$

So, Equation (17) results:

(17) $$\left(\theta_{A}-\frac{M_{A}}{k_{A}}\right)\;\;\;\;\;\;\;\;\;\;\;\;\;\;\;\left(\theta_{B}-\frac{M_{B}}{k_{B}}\right)$$

The relative rotation $\theta_{rA}$ and $\theta_{rB}$ of the beam ends in the effect of the springs. Taking into account some variables that depend exclusively on factors intrinsic to each spring (stiffness) and type of bar (length, section and moment of inertia). Now, it is possible to reformulate the above equations as follows, in Equations (18) and (19):

(18) $$M_{A}=\frac{E\cdot I}{L}\cdot \left(r_{ii}\cdot \theta_{A}+r_{ij}\cdot \theta_{B}\right)$$

(19) $$M_{B}=\frac{E\cdot I}{L}\cdot \left(r_{jj}\cdot \theta_{B}+r_{ij}\cdot \theta_{A}\right)$$

where:

$r_{ii}$, $r_{jj}$ and $r_{ij}$ are parameters that are solely dependent on bar and spring parameters.

Finally, the information needed to define the elemental vector of internal forces is now available. In this case, the most efficient method is to conduct a force analysis and include spring effects, elastic stiffness and the P-delta effect via the stiffness matrix. The updating of short-term moments and efforts (Equations (9), (10), (12) and (13)) is crucial because failure to include this updating in the defining code can result in convergence issues. With the nodal displacements and this matrix included, the total elemental vector of internal forces in matrixial format is:

(20) $$P_{int}^{e}\left\{\begin{array}{c} N_{i}\\ V_{i}\\ {\begin{array}{c} M_{i}\\ \begin{array}{c} N_{j}\\ V_{j}\\ M \end{array} \end{array}}_{j} \end{array}\right\}=\left[\left[k_{delta}\right]+\left[k_{semirig}\right]\right]\cdot \left[T_{transf}\right]\cdot \left\{\begin{array}{c} u_{i}\\ v_{i}\\ {\begin{array}{c} \theta_{i}\\ \begin{array}{c} u_{j}\\ v_{j}\\ \theta \end{array} \end{array}}_{j} \end{array}\right\}+P_{act}^{e}\left\{\begin{array}{c} 0\\ V_{A}\\ {\begin{array}{c} M_{A}\\ \begin{array}{c} 0\\ V_{B}\\ M \end{array} \end{array}}_{B} \end{array}\right\}\;$$

where:

$P_{int}^{e}$: Elemental force vector updated because of the effects of the springs and the P-delta effect;

$\left[k_{delta}\right]$: Non-linear matrix that includes stiffness due to the P-delta effect. The following is a detailed definition of the term:

(21) kdelta=NL·(000000065L100−65L100L102L2150−L10−L2300000000−65−L10065−L100L10−L2300−L102L215)

$\left[k_{semirig}\right]:$ The effect of the spring is included in this stiffness matrix. The Euler–Bernouilli stiffness is already included in this matrix. The following is a detailed definition of what we are talking about:

ksemirig=(E·AL00−E·AL000(rii+2·rij+rjj)·E·IL3(rii+rij)·E·IL20−(rii+2·rij+rjj)·E·IL3(rii+rjj)·E·IL20(rii+rij)·E·IL2rii·E·IL0−(rii+rij)·E·IL2rij·E·IL−E·AL00E·AL000−(rii+2·rij+rjj)·E·IL3−(rii+rij)·E·IL20(rii+2·rij+rjj)·E·IL3−(rij+rjj)·E·IL20(rij+rjj)·E·IL2rij·E·IL0−(rij+rjj)·E·IL2rjj·E·IL)

where:

$$r_{ii}=\frac{1}{k_{R}}\left(4+12\cdot \frac{E\cdot I}{L\cdot k_{B}}\right)\;\;\;\;\;\;\;\;\;\;\;\;\;\;\;\;\;\;\;\;\;\;\;\;\;\;\;\;\;\;\;\;r_{jj}=\frac{1}{k_{R}}\left(4+12\cdot \frac{E\cdot I}{L\cdot k_{A}}\right)$$

$$r_{ij}=\frac{2}{k_{R}}$$

$$k_{R}=\left(1+4\cdot \frac{E\cdot I}{L\cdot k_{A}}\right)\cdot \left(1+4\cdot \frac{E\cdot I}{L\cdot k_{B}}\right)-{\left(\frac{E\cdot I}{L}\right)}^{2}\cdot \left(\frac{4}{k_{A}\cdot k_{B}}\right)$$

All of these parameters are based on the previously defined spring stiffness spring stiffness *kA* and *kB*.

$\left[\left[k_{delta}\right]+\left[k_{semirig}\right]\right]$: We can refer to this matricidal total as the nonlinear stiffness matrix of a beam 2D element incorporating P-delta effects, which includes both the P-delta and springs effects;

$\left[T_{transf}\right]$: Rotation matrix whose only function is to influence the rotation related to the coordinates of the element in concordance with the coordinates of the global structure;

$P_{act}^{e}:$ Internal force vector updated as a result of the spring. As it has been previously stated, it does not contain axial forces.

### 2.3. Definition of the Tangent Stiffness Matrix

The vector of internal forces determines the tangent stiffness matrix. However, in cases when there are semi-rigid joints with non-linear matrices, the matrices are required to obtain the added stiffness or tangent stiffness matrix. The fact that the springs have a non-linear character necessitates the updating of this internal vector of forces, making the problem more difficult to solve. For this reason, the matrix is referred to as a tangent stiffness matrix, as it is required to linearize the residual equation in a nonlinear incremental method. If the elemental vector of forces (Equation (20)) is derived from the elemental displacements, the following result is obtained:

(22) $$K_{T}^{e}\left(d^{e}\right)=\frac{\partial p_{int}^{e}}{\partial d^{e}}=\int_{\Omega_{e}}^{}\frac{\partial B^{T}}{\partial d^{e}}\cdot \sigma d\Omega +\int_{\Omega_{e}}^{}B^{T}\frac{\partial \sigma }{\partial d^{e}}d\Omega$$

There is a geometric term and a material stiffness term. In this case, it is impossible to accept the possibility of smaller displacements, but it is reasonable to assume that the material is linear. As a result, the second term is equal to zero, leaving only the first. This first term refers to geometric stiffness; nevertheless, it is not linear due to two factors: the P-delta effect and the springs effect. As a result, the following equation is obtained:

(23) $$K_{T}^{e}\left(d^{e}\right)=\frac{\partial p_{int}^{e}}{\partial d^{e}}=\int_{\Omega_{e}}^{}\frac{\partial B^{T}}{\partial d^{e}}\cdot \sigma d\Omega =K_{\Delta }^{e}+K_{semirigid}^{e}$$

Without taking into account non-linear springs, the stiffness matrix responsible for the P-delta effect is the matrix P; the first of the terms is referred to as the “Stiffness geometrical matrix” [3,22]. The deductions mentioned in previous references are applied to the formulation of the matrix that includes this P effect. This geometric matrix is based on the axil effort, which is implemented using the form function for longitudinal deformation in a non-linear FEM environment.

Returning to the usual formulation, the matrix of stiffness in global coordinates is obtained by multiplying by a matrix of coordinates transformation:

(24) $$\left[k_{T}\right]={\left[T_{transf}\right]}^{T}\left[\left[K_{\Delta }^{e}\right]+\left[K_{semirigid}^{e}\right]\right]\cdot \left[T_{transf}\right]$$

This matrix will allow for the acquisition of new internal forces that can be compared to external forces, and whose reminder must converge to zero for some displacement increases. Once the problem has been solved, the matrix tangent solution to the problem is obtained, in accordance with the primary control method used (load control, or load and displacement, depending on the case). When it comes to coding, one thing to keep in mind is that the vector of internal forces, Pe, must be updated due to the non-linear effect of the springs. This vector of force update includes reversible moments and reversible efforts due to gravitational forces and spring effects, but not exiles. This is consistent with the effect of a transversal load superimposed on a beam with rotational springs, given that these springs do not generate axil effort. It is critical to incorporate these efforts’ updates because, on the contrary, the resolution of the beam–spring system using two nested Newton–Raphson equations does not converge.

### 2.4. No-Linear Rotary Spring Adjustment

To solve the non-linear problem and use rotational springs in structural systems with semi-rigid joints, it is required to find the balance function between the moment and the rotation for each of the load steps. Each step in the loading process necessitates its own adjustment within the overall control method that governs the process. The adjustment of springs necessitates iterative procedures for the adjustment of the added stiffness matrix and internal force vector Pe. The several iterations to be completed are referred to as sub-iterations in each iteration of this internal spring adjustment. General iterations are the iterations required to complete each step of the loading process. Each loading step, together with its general iterations, is governed by a general incremental method that differs from that of the springs. In essence, the problem is simple: adjusting the springs using a the Newton–Raphson method, selecting the appropriate initial values and correctly formulating the handling functions in the process code is all that is required. 

**Figure 8:**
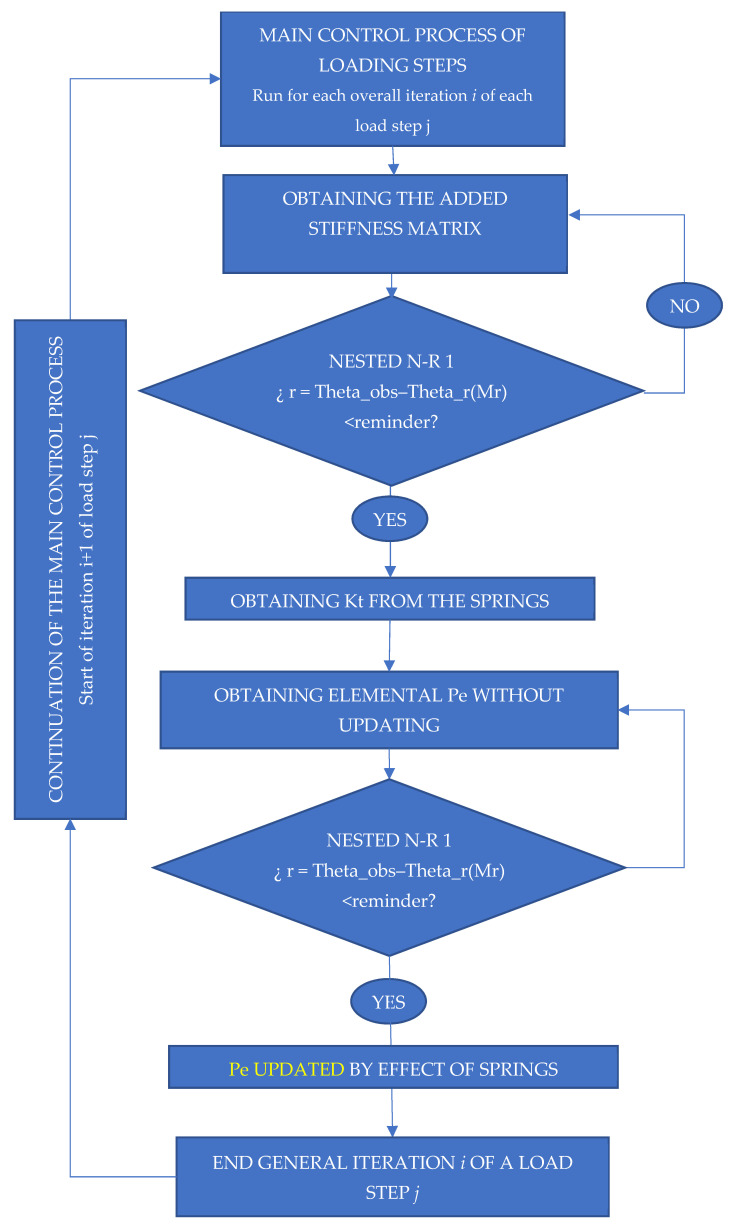
Flowchart. Nested N-R and principal N-R. Code implementation.

The springs’ added stiffness matrix and vector of forces have been adjusted and updated using two Newton–Raphson equations in series, each of which has been nested using the general incremental resolving method. This incremental generic method is completely independent of the formula and function of the two Newton–Raphson springs in series. The two series adjustments are required with the nested Newton–Raphson equation because they must be resolved inside each iteration general required to resolve each load step. There must be as many Newton–Raphson adjustments as each sub-iteration requires, as well as for each iteration of each loading step. All of this is based on a single Beam2D element. As a result, the adjustment process is depicted graphically in the following flow diagram in Figure 8.

Because the vector of internal forces Pe is updated by the effect of the springs and external loads, the two nested Newton–Raphson equations for the springs, in series with respect to the general control method, will not have the same income values.

The points to consider in the implementation of the two nested Newton–Raphson applications in series in one code are presented in a synthesis format, making the overall process of assembly of different elements in a nonlinear FEM known. This procedure is applicable to any type of semi-rigid mouldable union using non-linear rotational springs:

(1) Identifying the relative rotation generated by the springs (theta_r_observada, ec. 3 a 5);
(2) Obtaining the tangent stiffness matrix of each spring through derivation according to the increments in moment *Mr*(*A*) y *Mr*(*B*);
(3) Define the reminder as the difference between the observed and the calculated rotation;
(4) The results of stiffness for the springs are obtained by several iterations, for a generic iteration *i* of the loading dock *j*. The primary control method that oversees the process specifies the loading steps;
(5) Once the *Kt* of each spring has been determined, the elemental stiffness may be calculated. Without updating, a Pe is obtained. With this *Kt*, a vector of internal forces Pe is obtained, which has been updated due to the springs’ effect. This is now performed for each general iteration *i* of the loading process *j*.

Regarding the theta_r (Mr) rotation, it depends of the behaviour of the spring. In this case of study, it has been defined according the polynomial model of Fryes and Morris for type-4 joints. Particularly for these joints:

$$\mathrm{Theta}\_r\left(\mathrm{Mr}\right)=\left(\begin{array}{c} c1\cdot \mathrm{kappa}\cdot \mathrm{Mr}\left(A\right)+c2\cdot {\left(\mathrm{kappa}\cdot \mathrm{Mr}\left(A\right)\right)}^{3}+c3\cdot {\left(\mathrm{kappa}\cdot \mathrm{Mr}\left(A\right)\right)}^{5}\\ c1\cdot \mathrm{kappa}\cdot \mathrm{Mr}\left(B\right)+c2\cdot {\left(\mathrm{kappa}\cdot \mathrm{Mr}\left(B\right)\right)}^{3}+c3\cdot {\left(\mathrm{kappa}\cdot \mathrm{Mr}\left(B\right)\right)}^{5} \end{array}\right)$$

As a result of its derivation, the following is obtained:

$$K_{t}=\left(\begin{array}{c} c1\cdot \mathrm{kappa}+3\cdot c2*{\left(\mathrm{kappa}\cdot \mathrm{Mr}\left(A\right)\right)}^{2}+5\cdot c3\cdot {\left(\mathrm{kappa}\cdot \mathrm{Mr}\left(A\right)\right)}^{4}\;\;\;\;\;\;0\\ 0\;\;\;\;\;\;c1\cdot \mathrm{kappa}+3\cdot c2\cdot {\left(\mathrm{kappa}\cdot \mathrm{Mr}\left(B\right)\right)}^{2}+5\cdot c3\cdot {\left(\mathrm{kappa}\cdot \mathrm{Mr}\left(B\right)\right)}^{4} \end{array}\right)$$

## 3. Results and Discussion

**Figure 9:**
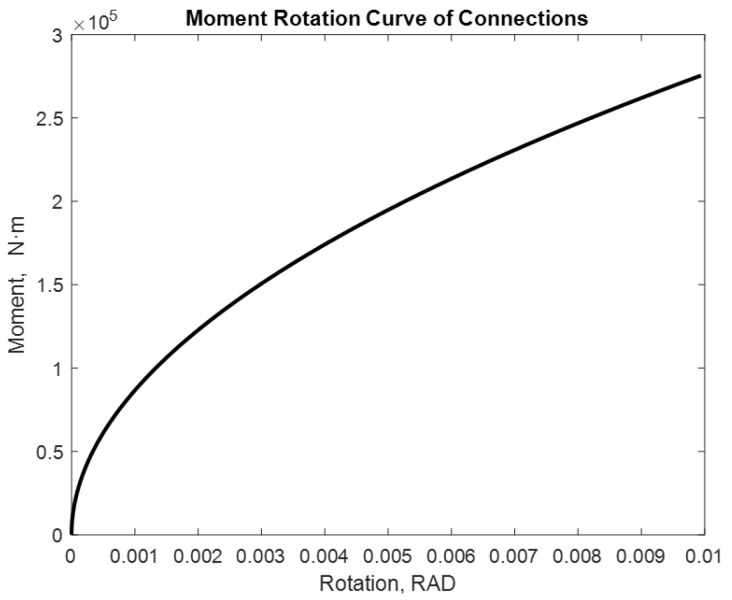
Rotation of 7.75 m in a beam with M = 275.40 kN·m. Beam2D element with two springs.

**Figure 10:**
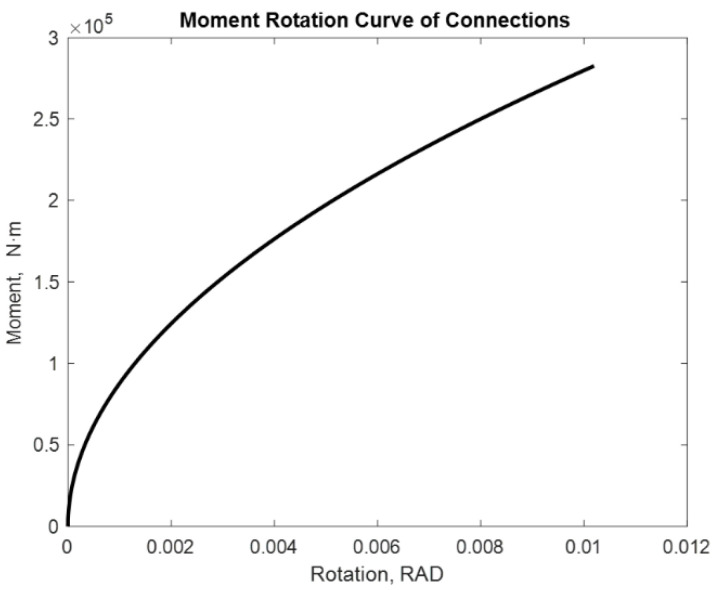
Rotation of 7.75 m in a beam with M = 282.42 kN·m Beam2D element with two springs.

**Figure 11:**
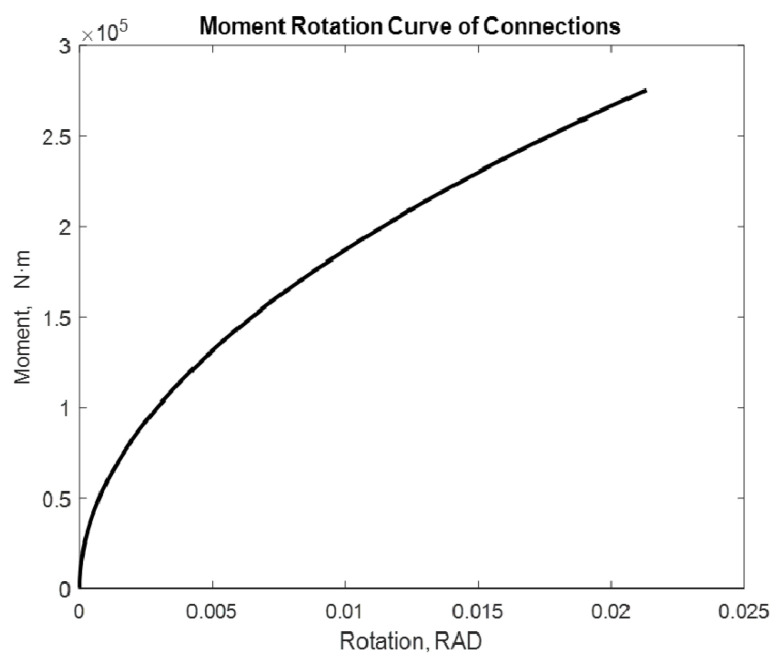
Rotation in a 7.75 m beam with M = 275.40 kN·m Beam2D element with 1 spring and cantilever.

**Figure 12:**
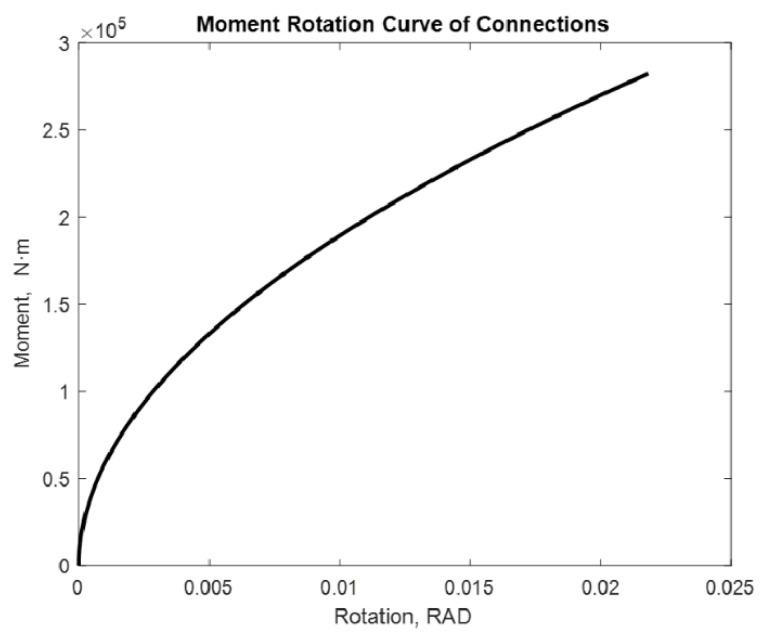
Rotation in 12 m beam with M = 282.42 kN·m Beam2D element with 1 spring and cantilever.

**Figure 13:**
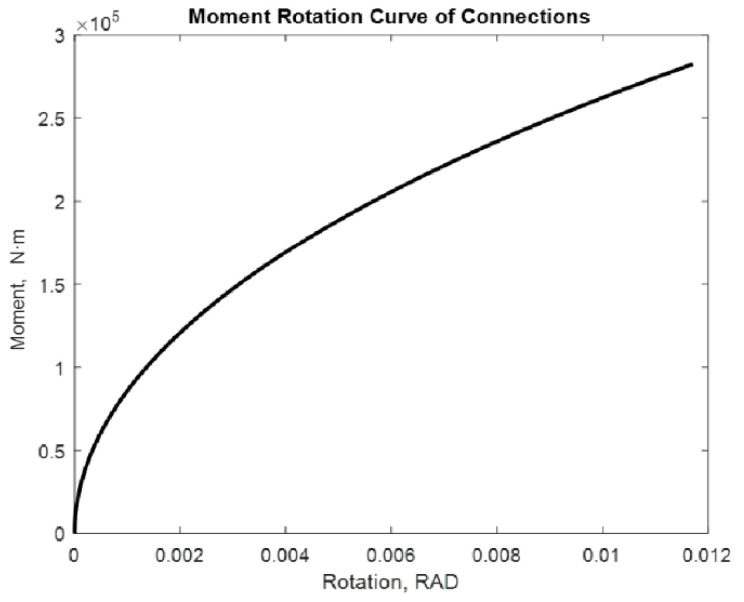
Rotation with p-delta effect for visualization. Axial compression.

**Figure 14:**
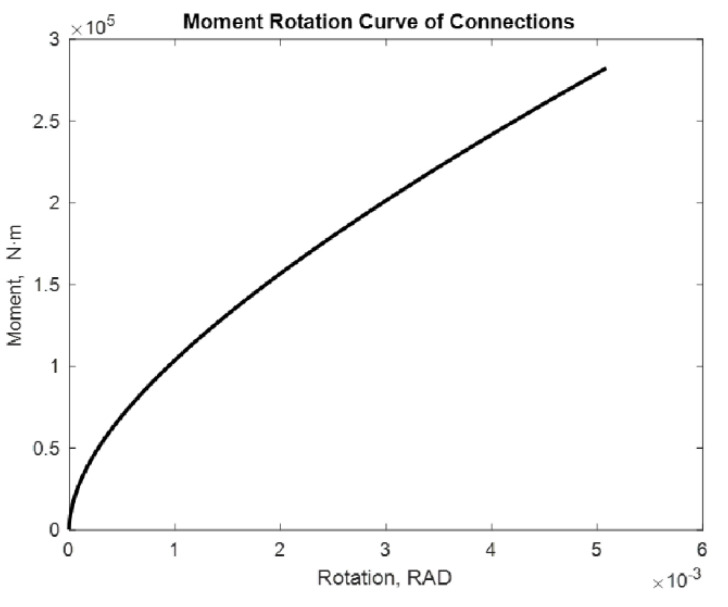
Rotation with an increased p-delta effect for visualization. Axial traction.

An analysis with various variants is carried out based on the previous foundations and a code according to the problem. The validity of the model has been confirmed by comparing the rotation diagrams for type-4 unions and similar loads from the models of Dhillon, B.S. and O’Malley, J.W., III [9] (Figure 9 and Table 1) and Degertekin, S.O. and Hayalioglu, M.S. [10] (Figure 1 and various tables), as well as the original model of Frye, M.J. (Figure 2 and Table 1). For the specific case, beams with given material characteristics and Fryes and Morris model values are examined for a semi-rigid connection with W 12 × 72 section and plate and bolts spacing for a semi-rigid type-4 connection (type-5 of Dhillon). The type-4 of Fryes and Morris is used when there are less deviations between the mathematical model and the experimental results. Table 1 is obtained:

The examined alternatives include: a beam with two semi-rigid joints in each of its extremities and a cantilevered beam with a semi-rigid joint in one of its extremities. Different loads with and without P-delta effect are applied. The distance between supports is considered constant with a lenght of 7.75 m. [17]. The moment values used are 275.40 kN·m and 282.42 kN·m. The following are the moment–rotation diagrams obtained using the proposed adjustment method. Figure 9, Figure 10, Figure 11, Figure 12, Figure 13 and Figure 14 show these results:

The maximum rotation obtained is 0.0099 rad. In the case of a moment equal to 282.42 kN·m.

We obtain 0.0102 rad. The curve maintains its slope, which is appropriate for this type of union; however, the rotation in this case increases as the moment increases.

If the process is repeated for a cantilevered beam at one of its extremities and a semi-rigid union at one of the moment’s extremities, the following result is obtained and shown in Figure 11 and Figure 12:

In this case, we obtain 0.0213 rad and 0.0218 rad rotations, respectively. In addition to increasing the rotation, increasing the moment enhances the rotation as well. However, the effect of the cantilever becomes apparent in the rotation, with a beam mounted on two supports increasing significantly. The order of the rotation is increasing. In this case, it is possible to observe that the curve is still the same.

It is possible to see in the previous diagrams, which correspond to semi-rigid joints, that the moment–rotation relationship is clearly not linear. Following that, it is possible to demonstrate the P-delta effect. To see this effect in greater detail, an amplification factor of ×10 to a 12 kN transversal traction load is applied. To see a better P-delta effect, turning on the option of increase obtains the following result for a moment of 282.42 kN·m.

Figure 13 and Figure 14 show the aforementioned results.

The rotation increases as a result of a loss of overall stiffness. Now, tracing efforts provide:

If the moment is aligned with an axial traction, the p-delta effect will effectively linearize the moment–rotation curve for the same initial conditions (load steps, applied load, etc). This implies that this effect has an impact on the tangential stiffness matrix. Despite the fact that the rotation must be interpreted qualitatively by the amplification factor used. The effect is an increase in the overall stiffness of the beam for tracing efforts accompanied by transverse load when this P-delta effect is assessed. In contrast, during compression efforts, the rotations increase, resulting in a reduction in the element’s overall stiffness and an increase in the rotation at its extremes. Although it is more crucial to consider this effect in columns, the beams may experience instabilities that will be more evident in the case of semi-rigid joints.

## 4. Conclusions

Following the completion of the work, it is possible to state that the Newton–Raphson method can be used to adjust and obtain moment–rotation diagrams that correlate to semi-rigid links. In the context of a nonlinear FEM, the model is implemented using rotationally nonlinear springs. According to the method presented, to adjust the tangential stiffness matrix and obtain the vector of internal forces, the use of two Newton–Raphson equations nested in series with the appropriate general control method is required. Furthermore, the moment–rotation relationship demonstrates a clear lack of geometric linearity. In the cases studied, the nonlinearity may be seen in beams with semi-rigid connections at both ends, as well as a cantilever; however, the order of the rotations differs. Small variations in the variables of a polynomial model might lead to convergence errors. In addition, proper initial value selection in Newton–Raphson nested equations facilitates convergence. Finally, it is possible to state that for tracing efforts, the P-delta effect entails a linearization of the moment–rotation curve, as well as a loss of stiffness and an increase in rotation in the case of compression axils. This coupling effect should be considered not just in columns but also in beam elements.

## Figures and Tables

**Table 1:** Connection type end-plate without column web stiffeners.

Properties of the Materials and Joints	Connection Type End-Plate without Column Web Stiffeners
Section	W 12 × 72
Plate thickness	tp = 2.54
Bolt spacing	dg = 72.48 mm
Bolt diameter	db = 2.54

## Data Availability

Data are contained within the article.
